# Supplementary material for: Is Fasting Superior to Continuous Caloric Restriction for Weight Loss and Metabolic Outcomes in Obese Adults? A Systematic Review and Meta-Analysis of Randomized Clinical Trials
Source: Nutrients. 2024 Oct 18;16(20):3533. doi: 10.3390/nu16203533 (PMC11510157; doi:10.3390/nu16203533)
Supplement: Supplementary file 1 [file nutrients-16-03533-s001.zip › nutrients-3252659-supplementary.pdf]

Short-term effects (< 6 months) in body lean mass

Study or Subgroup	Intermittent Fasting Mean	SD	Total	Caloric Restriction Mean	SD	Total	Weight	Mean Difference IV, Fixed, 95% CI
Hutchison AT 2019	-1.4	1.41	18	-0.6	1.47	22	21.4%	-0.80 [-1.70, 0.10]
Lin S 2023	-0.12	2.35	26	-0.23	2.25	25	10.8%	0.11 [-1.15, 1.37]
Liu D 2022	-1.9	2.12	69	-1.7	2.13	70	34.4%	-0.20 [-0.91, 0.51]
Steger FL 2021	-0.65	5.94	18	-1.7	2.13	17	2.0%	1.05 [-1.87, 3.97]
Steger FL 2023	-1.12	1.28	15	-1.75	4.27	21	4.6%	0.63 [-1.31, 2.57]
Thomas EA 2022	-1.5	1.4	32	-1.1	1.8	31	26.9%	-0.40 [-1.20, 0.40]
**Total (95% CI)**			**178**			**186**	**100.0%**	**-0.29 [-0.70, 0.13]**

Heterogeneity: Chi² = 3.44, df = 5 (P = 0.63); I² = 0%
Test for overall effect: Z = 1.35 (P = 0.18)

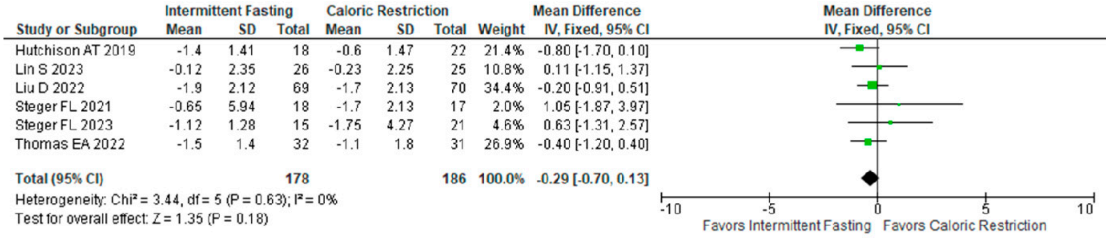

Long-term effects (> 6 months) in body lean mass

Study or Subgroup	Intermittent Fasting Mean	SD	Total	Caloric Restriction Mean	SD	Total	Weight	Mean Difference IV, Fixed, 95% CI
Lin S 2023	-0.41	1.33	26	-0.74	1.89	25	33.3%	0.33 [-0.57, 1.23]
Liu D 2022	-1.7	2.54	69	-1.4	2.35	70	40.7%	-0.30 [-1.11, 0.51]
Thomas EA 2022	-1.2	2.3	32	-1.5	1.8	31	26.0%	0.30 [-0.72, 1.32]
**Total (95% CI)**			**127**			**126**	**100.0%**	**0.07 [-0.45, 0.58]**

Heterogeneity: Chi² = 1.31, df = 2 (P = 0.52); I² = 0%
Test for overall effect: Z = 0.25 (P = 0.80)

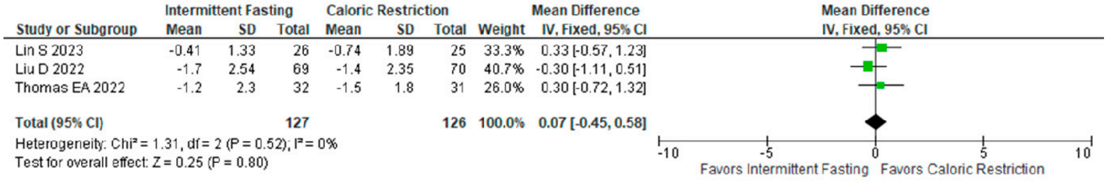

Figure S1. Forest plot of the short- (< 6 months) and long-term (> 6 months) effects of fasting based interventions versus continuous caloric restriction on body lean mass.

Short-term effects (< 6 months) in body fat mass

Study or Subgroup	Intermittent Fasting Mean	SD	Total	Caloric Restriction Mean	SD	Total	Weight	Mean Difference IV, Fixed, 95% CI
Hutchison AT 2019	-3.9	1.88	18	-2.8	1.96	22	21.2%	-1.10 [-2.29, 0.09]
Lin S 2023	-2.68	6.64	26	-2.25	6.36	25	2.4%	-0.43 [-4.00, 3.14]
Liu D 2022	-6.9	4.87	69	-6.4	4.7	70	11.9%	-0.50 [-2.09, 1.09]
Pinto AM 2020	-2.23	3.51	21	0.89	3.59	22	6.7%	-3.12 [-5.24, -1.00]
Steger FL 2021	-8.1	5.42	18	-9.12	7.21	17	1.7%	1.02 [-3.22, 5.26]
Steger FL 2023	-5.9	4.53	15	-3.1	3.94	21	3.7%	-2.80 [-5.65, 0.05]
Teong TX 2021	-3.9	1.7	22	-2.8	1.9	24	27.9%	-1.10 [-2.14, -0.06]
Thomas EA 2022	-2.8	1.8	32	-2.1	2.6	31	24.6%	-0.70 [-1.81, 0.41]
**Total (95% CI)**			**221**			**232**	**100.0%**	**-1.08 [-1.63, -0.53]**

Heterogeneity: Chi² = 6.99, df = 7 (P = 0.43); I² = 0%
Test for overall effect: Z = 3.84 (P = 0.0001)

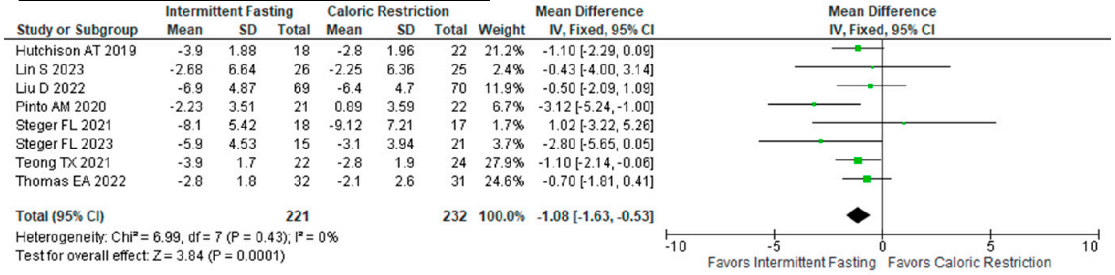

Long-term effects (> 6 months) in body fat mass

Study or Subgroup	Intermittent Fasting Mean	SD	Total	Caloric Restriction Mean	SD	Total	Weight	Mean Difference IV, Fixed, 95% CI
de Oliveira 2021	-0.21	5.79	31	0.3	5.12	27	16.2%	-0.51 [-3.32, 2.30]
Lin S 2023	-2.2	4.5	26	-2.61	8.98	25	8.3%	0.41 [-3.51, 4.33]
Liu D 2022	-5.9	5.09	69	-4.5	4.91	70	46.1%	-1.40 [-3.06, 0.26]
Thomas EA 2022	-3.5	4	32	-2.6	4.4	31	29.5%	-0.90 [-2.98, 1.18]
**Total (95% CI)**			**158**			**153**	**100.0%**	**-0.96 [-2.09, 0.17]**

Heterogeneity: Chi² = 0.84, df = 3 (P = 0.84); I² = 0%
Test for overall effect: Z = 1.66 (P = 0.10)

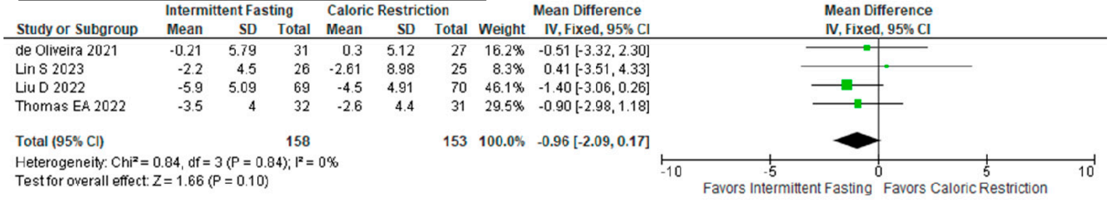

Figure S2. Forest plot of the short- (< 6 months) and long-term (> 6 months) effects of fasting based interventions versus continuous caloric restriction on body fat mass.

(A) **Short-term effects (< 6 months) in waist circumference**

Study or Subgroup	Intermittent Fasting Mean	SD	Total	Caloric Restriction Mean	SD	Total	Weight	Mean Difference IV, Random, 95% CI
Hutchison AT 2019	-7.6	5.63	18	-5.2	4.9	22	11.6%	-2.40 [-5.71, 0.91]
Lin S 2023	-5.54	8.55	26	-4.69	8.19	25	7.9%	-0.85 [-5.44, 3.74]
Liu D 2022	-9.4	6.57	69	-8.7	6.19	70	16.4%	-0.70 [-2.82, 1.42]
Pinto AM 2020	-3	2.05	21	-6	3.45	22	18.4%	3.00 [1.31, 4.69]
Steger FL 2021	-8.08	10.8	18	-8.9	8.3	17	4.9%	0.82 [-5.54, 7.18]
Steger FL 2023	-4.52	4.07	15	-6.21	6.43	21	11.1%	1.69 [-1.75, 5.13]
Sundfør TM 2018	-8	5.6	54	-9.2	5.4	58	16.8%	1.20 [-0.84, 3.24]
Teong TX 2021	-7.6	5.4	22	-5.2	4.7	24	12.9%	-2.40 [-5.34, 0.54]
**Total (95% CI)**			**243**			**259**	**100.0%**	**0.21 [-1.38, 1.80]**

Heterogeneity: Tau² = 2.85; Chi² = 17.44, df = 7 (P = 0.01); I² = 60%
Test for overall effect: Z = 0.26 (P = 0.80)

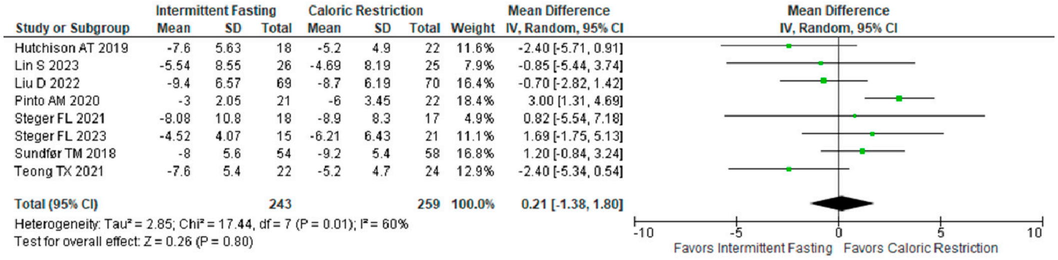

(B) **Short-term effects (< 6 months) in hip circumference**

Study or Subgroup	Intermittent Fasting Mean	SD	Total	Caloric Restriction Mean	SD	Total	Weight	Mean Difference IV, Fixed, 95% CI
Hutchison AT 2019	-5.3	3.75	18	-4.4	4.9	22	23.4%	-0.90 [-3.58, 1.78]
Steger FL 2021	-10.3	6.17	18	-9.97	12.2	17	4.0%	-0.33 [-6.79, 6.13]
Sundfør TM 2018	-6.8	4.6	54	-7.5	5.9	58	44.2%	0.70 [-1.25, 2.65]
Teong TX 2021	-5.4	3.7	22	-4.4	4.7	24	28.4%	-1.00 [-3.43, 1.43]
**Total (95% CI)**			**112**			**121**	**100.0%**	**-0.20 [-1.50, 1.10]**

Heterogeneity: Chi² = 1.49, df = 3 (P = 0.68); I² = 0%
Test for overall effect: Z = 0.30 (P = 0.76)

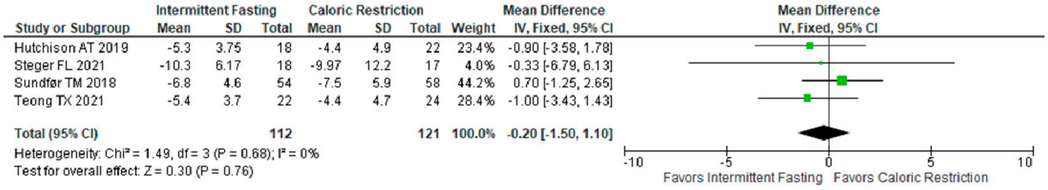

(C) **Long-term effects (> 6 months) in waist circumference**

Study or Subgroup	Intermittent Fasting Mean	SD	Total	Caloric Restriction Mean	SD	Total	Weight	Mean Difference IV, Random, 95% CI
de Oliveira 2021	-2.96	11.9	31	-0.11	9.8	27	12.0%	-2.85 [-8.44, 2.74]
Lin S 2023	-6.44	5.91	26	-3.77	9.88	25	16.4%	-2.67 [-7.16, 1.82]
Liu D 2022	-8.8	6.99	69	-7	6.62	70	32.8%	-1.80 [-4.06, 0.46]
Sundfør TM 2018	-8	4	54	-9.2	5	58	38.8%	1.20 [-0.47, 2.87]
**Total (95% CI)**			**180**			**180**	**100.0%**	**-0.90 [-3.12, 1.32]**

Heterogeneity: Tau² = 2.58; Chi² = 6.64, df = 3 (P = 0.08); I² = 55%
Test for overall effect: Z = 0.80 (P = 0.42)

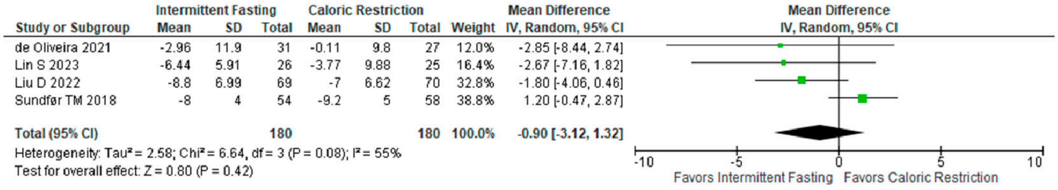

Figure S3. (A) Forest plot of the short-term effects (< 6 months) of fasting based interventions versus continuous caloric restriction on waist circumference. (B) Forest plot of the short-term effects (< 6 months) of fasting based interventions versus continuous caloric restriction on hip circumference. (C) Forest plot of the long-term effects (> 6 months) of fasting based interventions versus continuous caloric restriction on waist circumference.

Short-term effects (< 6 months) in systolic blood pressure

Study or Subgroup	Intermittent Fasting Mean	SD	Total	Caloric Restriction Mean	SD	Total	Weight	Mean Difference IV, Fixed, 95% CI
Hutchison AT 2019	-0.6	15.01	18	-4.2	14.21	22	5.3%	3.60 [-5.53, 12.73]
Lin S 2023	-0.59	12.37	26	-5.54	12.36	25	9.5%	4.95 [-1.84, 11.74]
Liu D 2022	-10.1	9.75	69	-8.1	9.39	70	43.2%	-2.00 [-5.18, 1.18]
Steger FL 2021	-6.78	6.61	18	-7.44	13.9	17	8.3%	0.66 [-6.62, 7.94]
Steger FL 2023	-9.08	8.06	15	-2.95	9.24	21	13.6%	-6.13 [-11.81, -0.45]
Sundfør TM 2018	-4.9	14.1	54	-5.8	10.7	58	20.2%	0.90 [-3.76, 5.56]
**Total (95% CI)**			**200**			**213**	**100.0%**	**-0.80 [-2.89, 1.29]**

Heterogeneity: Chi² = 8.24, df = 5 (P = 0.14); I² = 39%
Test for overall effect: Z = 0.75 (P = 0.45)

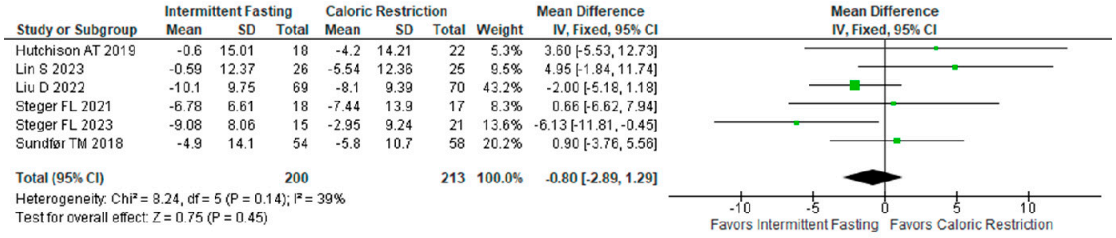

Long-term effects (> 6 months) in systolic blood pressure

Study or Subgroup	Intermittent Fasting Mean	SD	Total	Caloric Restriction Mean	SD	Total	Weight	Mean Difference IV, Fixed, 95% CI
de Oliveira 2021	-1.69	16.15	31	-4.59	10.04	27	11.7%	2.90 [-3.93, 9.73]
Lin S 2023	-1.78	13.44	26	-4.62	11.53	25	11.6%	2.84 [-4.02, 9.70]
Liu D 2022	-8.1	9.96	69	-7.7	10.03	70	49.4%	-0.40 [-3.72, 2.92]
Sundfør TM 2018	-1.9	12.3	54	-3.6	11.8	58	27.3%	1.70 [-2.77, 6.17]
**Total (95% CI)**			**180**			**180**	**100.0%**	**0.93 [-1.40, 3.27]**

Heterogeneity: Chi² = 1.35, df = 3 (P = 0.72); I² = 0%
Test for overall effect: Z = 0.78 (P = 0.43)

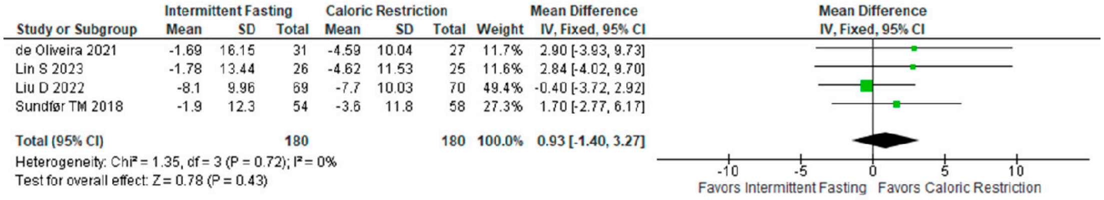

Figure S4. Forest plot of the short- (< 6 months) and long-term (> 6 months) effects of fasting based interventions versus continuous caloric restriction on systolic blood pressure.

Short-term effects (< 6 months) in diastolic blood pressure

Study or Subgroup	Intermittent Fasting Mean	SD	Total	Caloric Restriction Mean	SD	Total	Weight	Mean Difference IV, Fixed, 95% CI
Hutchison AT 2019	-0.4	4.69	25	-0.1	7.35	26	16.6%	-0.30 [-3.67, 3.07]
Lin S 2023	-1.71	8.4	30	-0.33	8.77	30	10.0%	-1.38 [-5.73, 2.97]
Liu D 2022	-6	7.63	69	-5.1	7.47	70	30.0%	-0.90 [-3.41, 1.61]
Steger FL 2021	-3.03	7.75	18	-4.32	6.43	17	8.5%	1.29 [-3.42, 6.00]
Steger FL 2023	-2.5	5.39	29	-6	10.77	30	10.1%	3.50 [-0.82, 7.82]
Sundfør TM 2018	-5.8	7.5	54	-4.7	7.4	58	24.8%	-1.10 [-3.86, 1.66]
**Total (95% CI)**			**225**			**231**	**100.0%**	**-0.27 [-1.64, 1.11]**

Heterogeneity: Chi² = 4.18, df = 5 (P = 0.52); I² = 0%
Test for overall effect: Z = 0.38 (P = 0.70)

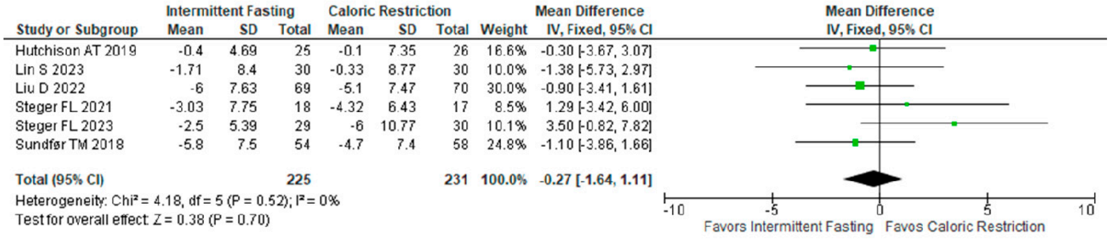

Long-term effects (> 6 months) in diastolic blood pressure

Study or Subgroup	Intermittent Fasting Mean	SD	Total	Caloric Restriction Mean	SD	Total	Weight	Mean Difference IV, Fixed, 95% CI
de Oliveira 2021	-2.4	11.59	31	-4.22	10.73	27	9.1%	1.82 [-3.93, 7.57]
Lin S 2023	-0.82	10.38	26	0.99	8.34	25	11.3%	-1.81 [-6.97, 3.35]
Liu D 2022	-5.1	8.48	69	-3.8	7.9	70	40.5%	-1.30 [-4.03, 1.43]
Sundfør TM 2018	-3	7.3	54	-2.9	7.7	58	39.0%	-0.10 [-2.88, 2.68]
**Total (95% CI)**			**180**			**180**	**100.0%**	**-0.60 [-2.34, 1.13]**

Heterogeneity: Chi² = 1.27, df = 3 (P = 0.74); I² = 0%
Test for overall effect: Z = 0.68 (P = 0.49)

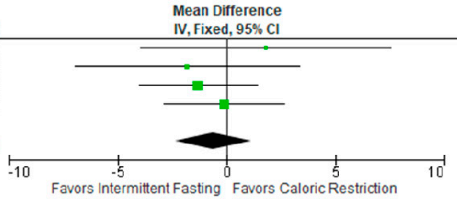

Figure S5. Forest plot of the short- (< 6 months) and long-term (> 6 months) effects of fasting based interventions versus continuous caloric restriction on diastolic blood pressure.

Short-term effects (< 6 months) in HDL-Cholesterol

Study or Subgroup	Intermittent Fasting Mean	SD	Total	Caloric Restriction Mean	SD	Total	Weight	Mean Difference IV, Fixed, 95% CI
Hutchison AT 2019	-3.87	5.44	18	-1.93	3.79	22	21.9%	-1.94 [-4.91, 1.03]
Lin S 2023	-1.78	9.11	26	1.01	11.19	25	6.1%	-2.79 [-8.40, 2.82]
Liu D 2022	4.2	7.84	69	2.7	7.47	70	29.8%	1.50 [-1.05, 4.05]
Steger FL 2023	-5.84	8.17	15	-1.74	10.51	21	5.2%	-4.10 [-10.21, 2.01]
Sundfør TM 2018	1.93	7.73	54	2.32	3.87	58	36.9%	-0.39 [-2.68, 1.90]
**Total (95% CI)**			**182**			**196**	**100.0%**	**-0.51 [-1.90, 0.89]**

Heterogeneity: Chi² = 5.26, df = 4 (P = 0.26); I² = 24%
Test for overall effect: Z = 0.71 (P = 0.48)

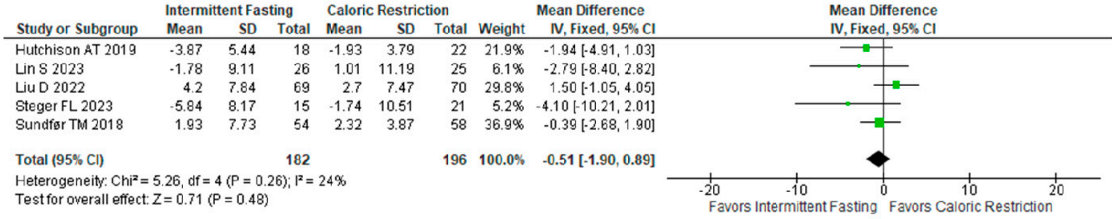

Long-term effects (> 6 months) in HDL-Cholesterol

Study or Subgroup	Intermittent Fasting Mean	SD	Total	Caloric Restriction Mean	SD	Total	Weight	Mean Difference IV, Fixed, 95% CI
Lin S 2023	-1.53	7.97	26	-1.53	7.97	25	24.2%	0.00 [-4.38, 4.38]
Liu D 2022	4.6	8.26	69	2.9	7.9	70	64.2%	1.70 [-0.99, 4.39]
Sundfør TM 2018	5.03	7.73	54	5.03	23.2	58	11.6%	0.00 [-6.32, 6.32]
**Total (95% CI)**			**149**			**153**	**100.0%**	**1.09 [-1.06, 3.24]**

Heterogeneity: Chi² = 0.55, df = 2 (P = 0.76); I² = 0%
Test for overall effect: Z = 0.99 (P = 0.32)

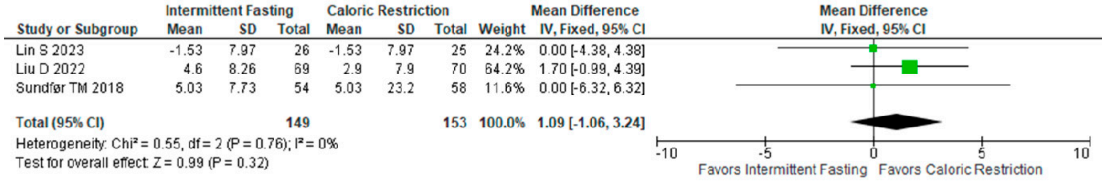

Figure S6. Forest plot of the short- (< 6 months) and long-term (> 6 months) effects of fasting based interventions versus continuous caloric restriction on HDL-cholesterol.

Short-term effects (< 6 months) in LDL-Cholesterol

Study or Subgroup	Intermittent Fasting Mean	Intermittent Fasting SD	Intermittent Fasting Total	Caloric Restriction Mean	Caloric Restriction SD	Caloric Restriction Total	Weight	Mean Difference IV, Random, 95% CI
Hutchison AT 2019	-14.31	12.7	18	-5.03	15.16	22	22.5%	-9.28 [-17.91, -0.65]
Lin S 2023	0.7	21.32	26	-6	25.08	25	16.5%	6.70 [-6.10, 19.50]
Liu D 2022	-5.9	25.22	69	-11.3	24.54	70	23.0%	5.40 [-2.87, 13.67]
Steger FL 2023	-15.9	25.95	15	-6.46	24.48	21	12.2%	-9.44 [-26.24, 7.36]
Sundfør TM 2018	-6.19	15.47	54	2.71	19.34	58	25.9%	-8.90 [-15.37, -2.43]
**Total (95% CI)**			**182**			**196**	**100.0%**	**-3.19 [-10.61, 4.22]**

Heterogeneity: Tau² = 44.37; Chi² = 11.69, df = 4 (P = 0.02); I² = 66%
Test for overall effect: Z = 0.84 (P = 0.40)

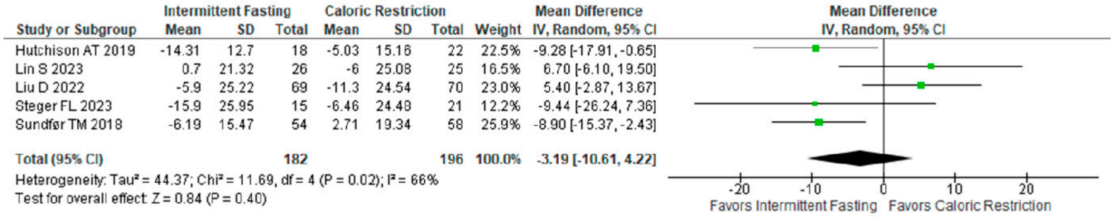

Long-term effects (> 6 months) in LDL-Cholesterol

Study or Subgroup	Intermittent Fasting Mean	Intermittent Fasting SD	Intermittent Fasting Total	Caloric Restriction Mean	Caloric Restriction SD	Caloric Restriction Total	Weight	Mean Difference IV, Fixed, 95% CI
Lin S 2023	-0.86	22.17	26	-1.33	18.44	25	24.9%	0.47 [-10.21, 11.15]
Liu D 2022	-8.4	26.7	69	-8.9	26.25	70	36.7%	0.50 [-8.30, 9.30]
Sundfør TM 2018	-1.16	23.2	54	3.09	23.2	58	38.4%	-4.25 [-12.85, 4.35]
**Total (95% CI)**			**149**			**153**	**100.0%**	**-1.33 [-6.66, 4.00]**

Heterogeneity: Chi² = 0.72, df = 2 (P = 0.70); I² = 0%
Test for overall effect: Z = 0.49 (P = 0.62)

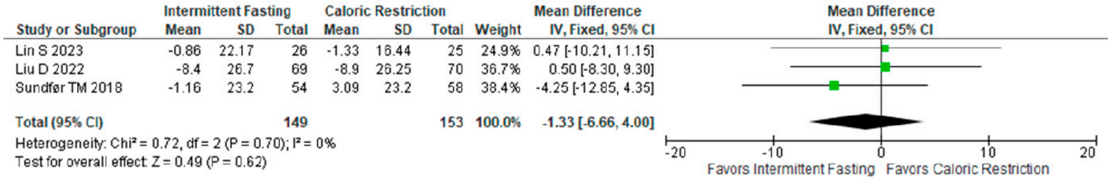

Figure S7. Forest plot of the short- (< 6 months) and long-term (> 6 months) effects of fasting based interventions versus continuous caloric restriction on LDL-cholesterol.

Short-term effects (< 6 months) in total cholesterol

Study or Subgroup	Intermittent Fasting Mean	Intermittent Fasting SD	Intermittent Fasting Total	Caloric Restriction Mean	Caloric Restriction SD	Caloric Restriction Total	Weight	Mean Difference IV, Random, 95% CI
Hutchison AT 2019	-22.82	14.52	18	-9.28	18.96	22	15.7%	-13.54 [-23.92, -3.16]
Lin S 2023	-1.09	20.89	26	-4.69	27.22	25	11.7%	3.60 [-9.75, 16.95]
Liu D 2022	-9	29.03	69	-13.7	27.75	70	17.3%	4.70 [-4.74, 14.14]
Pinto AM 2020	-5.8	7.73	21	-6	3.35	22	29.2%	0.20 [-3.39, 3.79]
Steger FL 2023	-24.85	34.66	15	-10.68	28	21	5.9%	-14.17 [-35.41, 7.07]
Sundfør TM 2018	-10.05	23.2	54	-2.71	19.34	58	20.1%	-7.34 [-15.28, 0.60]
**Total (95% CI)**			**203**			**218**	**100.0%**	**-3.15 [-8.84, 2.54]**

Heterogeneity: Tau² = 25.53; Chi² = 11.69, df = 5 (P = 0.04); I² = 57%
Test for overall effect: Z = 1.08 (P = 0.28)

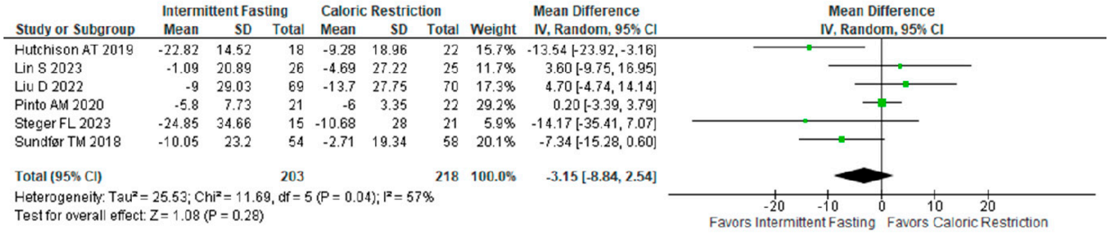

Long-term effects (> 6 months) in total cholesterol

Study or Subgroup	Intermittent Fasting Mean	Intermittent Fasting SD	Intermittent Fasting Total	Caloric Restriction Mean	Caloric Restriction SD	Caloric Restriction Total	Weight	Mean Difference IV, Fixed, 95% CI
Lin S 2023	-1.69	21.54	26	-1.64	21.85	25	25.4%	-0.05 [-11.96, 11.86]
Liu D 2022	-7.3	29.67	69	-9.3	28.39	70	38.7%	2.00 [-7.66, 11.66]
Sundfør TM 2018	2.71	27.07	54	6.57	27.07	58	35.9%	-3.86 [-13.89, 6.17]
**Total (95% CI)**			**149**			**153**	**100.0%**	**-0.62 [-6.63, 5.39]**

Heterogeneity: Chi² = 0.69, df = 2 (P = 0.71); I² = 0%
Test for overall effect: Z = 0.20 (P = 0.84)

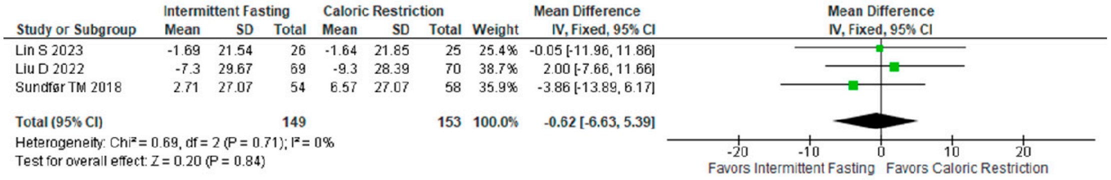

Figure S8. Forest plot of the short- (< 6 months) and long-term (> 6 months) effects of fasting based interventions versus continuous caloric restriction on total cholesterol.

Short-term effects (< 6 months) in triglycerides

Study or Subgroup	Intermittent Fasting Mean	SD	Total	Caloric Restriction Mean	SD	Total	Weight	Mean Difference IV, Fixed, 95% CI
Hutchison AT 2019	-21.26	29.08	18	-14.17	21.7	22	36.5%	-7.09 [-23.30, 9.12]
Lin S 2023	0.15	62.53	26	6.84	32.83	25	12.9%	-6.69 [-33.95, 20.57]
Liu D 2022	-44.8	64.63	69	-31.7	63.6	70	21.1%	-13.10 [-34.42, 8.22]
Steger FL 2023	-14.66	43.3	15	-6.71	58.25	21	8.7%	-7.95 [-41.13, 25.23]
Sundfør TM 2018	-30	62	54	-31.89	53.14	58	20.8%	1.89 [-19.57, 23.35]
**Total (95% CI)**			**182**			**196**	**100.0%**	**-6.51 [-16.30, 3.28]**

Heterogeneity: Chi² = 0.97, df = 4 (P = 0.91); I² = 0%
Test for overall effect: Z = 1.30 (P = 0.19)

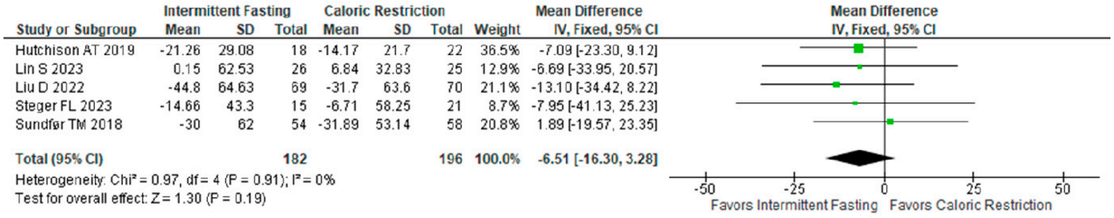

Long-term effects (> 6 months) in triglycerides

Study or Subgroup	Intermittent Fasting Mean	SD	Total	Caloric Restriction Mean	SD	Total	Weight	Mean Difference IV, Fixed, 95% CI
Lin S 2023	3.6	37.01	26	-0.02	34.36	25	42.8%	3.62 [-15.97, 23.21]
Liu D 2022	-25.5	70.78	69	-19.6	69.37	70	30.3%	-5.90 [-29.20, 17.40]
Sundfør TM 2018	-27.45	70.86	54	-9.74	61.99	58	26.9%	-17.71 [-42.44, 7.02]
**Total (95% CI)**			**149**			**153**	**100.0%**	**-5.00 [-17.82, 7.83]**

Heterogeneity: Chi² = 1.76, df = 2 (P = 0.41); I² = 0%
Test for overall effect: Z = 0.76 (P = 0.45)

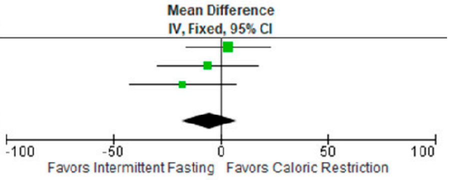

Figure S9. Forest plot of the short- (< 6 months) and long-term (> 6 months) effects of fasting based interventions versus continuous caloric restriction on triglycerides.

Short-term effects (< 6 months) in fasting glucose

Study or Subgroup	Intermittent Fasting Mean	SD	Total	Caloric Restriction Mean	SD	Total	Weight	Mean Difference IV, Random, 95% CI
Hutchison AT 2019	-3.6	8.45	18	1.8	8.82	22	17.3%	-5.40 [-10.77, -0.03]
Lin S 2023	-1.65	13.68	26	-0.81	12.88	25	14.4%	-0.84 [-8.13, 6.45]
Liu D 2022	-5	14.62	69	-4.1	14.3	70	18.2%	-0.90 [-5.71, 3.91]
Pinto AM 2020	0.72	2.88	21	-4.5	2.34	22	22.2%	5.22 [3.65, 6.79]
Steger FL 2023	-15.67	16.54	15	-6.65	14.82	21	10.3%	-9.02 [-19.52, 1.48]
Sundfør TM 2018	-3.6	16.2	54	-3.6	10.8	58	17.7%	0.00 [-5.14, 5.14]
**Total (95% CI)**			**203**			**218**	**100.0%**	**-0.99 [-5.54, 3.56]**

Heterogeneity: Tau² = 23.63; Chi² = 26.85, df = 5 (P < 0.0001); I² = 81%
Test for overall effect: Z = 0.43 (P = 0.67)

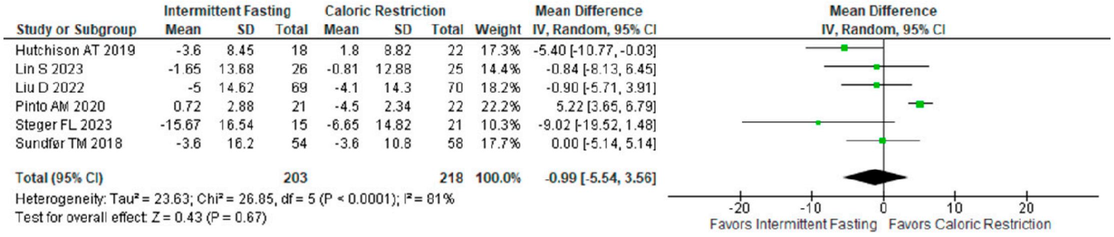

Long-term effects (> 6 months) in fasting glucose

Study or Subgroup	Intermittent Fasting Mean	SD	Total	Caloric Restriction Mean	SD	Total	Weight	Mean Difference IV, Fixed, 95% CI
Lin S 2023	2.82	10.63	26	5.83	12.25	25	29.5%	-3.01 [-9.32, 3.30]
Liu D 2022	-3.5	17.38	69	-3	15.79	70	38.5%	-0.50 [-6.02, 5.02]
Sundfør TM 2018	-3.6	21.62	54	0	7.21	58	32.0%	-3.60 [-9.66, 2.46]
**Total (95% CI)**			**149**			**153**	**100.0%**	**-2.23 [-5.66, 1.19]**

Heterogeneity: Chi² = 0.63, df = 2 (P = 0.73); I² = 0%
Test for overall effect: Z = 1.28 (P = 0.20)

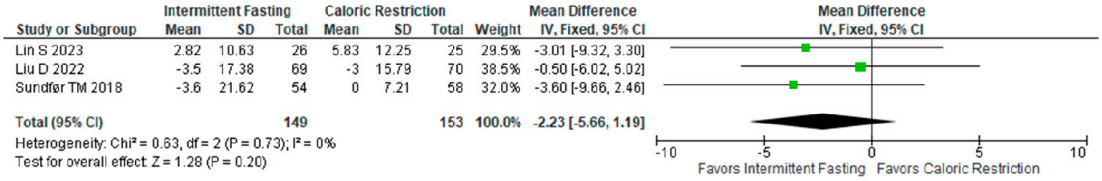

Figure S10. Forest plot of the short- (< 6 months) and long-term (> 6 months) effects of fasting based interventions versus continuous caloric restriction on fasting glucose

(A) Short-term effects (< 6 months) in fasting insulin

Study or Subgroup	Intermittent Fasting Mean	SD	Total	Caloric Restriction Mean	SD	Total	Weight	Mean Difference IV, Fixed, 95% CI
Hutchison AT 2019	-25	32.58	18	-8.33	37.42	22	8.5%	-16.67 [-38.37, 5.03]
Lin S 2023	-20.07	62.58	30	-6.32	39.51	30	5.7%	-13.75 [-40.23, 12.73]
Pinto AM 2020	-9.6	15.96	21	-6.06	14.22	22	48.6%	-3.54 [-12.59, 5.51]
Steger FL 2023	-9.53	13.98	15	0	17.64	21	37.2%	-9.53 [-19.87, 0.81]
**Total (95% CI)**			**84**			**95**	**100.0%**	**-7.46 [-13.77, -1.15]**

Heterogeneity: $Chi^2 = 1.78$, df = 3 (P = 0.62); $I^2 = 0\%$
Test for overall effect: Z = 2.32 (P = 0.02)

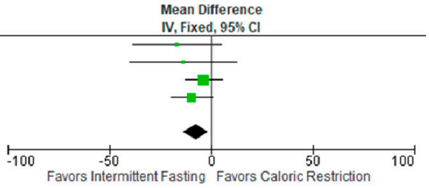

(B) Short-term effects (< 6 months) in HbA1C

Study or Subgroup	Intermittent Fasting Mean	SD	Total	Caloric Restriction Mean	SD	Total	Weight	Mean Difference IV, Fixed, 95% CI
Lin S 2023	0	0.39	26	-0.05	0.32	25	25.1%	0.05 [-0.15, 0.25]
Steger FL 2023	-0.08	0.23	15	-0.02	0.23	21	41.2%	-0.06 [-0.21, 0.09]
Sundfør TM 2018	-0.3	0.5	54	-0.2	0.4	58	33.7%	-0.10 [-0.27, 0.07]
**Total (95% CI)**			**95**			**104**	**100.0%**	**-0.05 [-0.14, 0.05]**

Heterogeneity: $Chi^2 = 1.35$, df = 2 (P = 0.51); $I^2 = 0\%$
Test for overall effect: Z = 0.92 (P = 0.36)

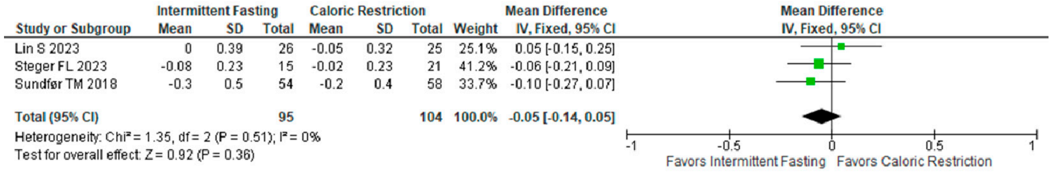

(C) Short-term effects (< 6 months) in HOMA-IR

Study or Subgroup	Intermittent Fasting Mean	SD	Total	Caloric Restriction Mean	SD	Total	Weight	Mean Difference IV, Fixed, 95% CI
Hutchison AT 2019	-0.9	1.41	18	-0.4	1.47	22	2.2%	-0.50 [-1.40, 0.40]
Lin S 2023	-0.66	2.65	26	-0.28	1.47	25	1.3%	-0.38 [-1.55, 0.79]
Liu D 2022	-1.4	2.33	69	-1.2	2.56	70	2.6%	-0.20 [-1.01, 0.61]
Pinto AM 2020	-0.42	0.26	21	-0.3	0.19	22	93.7%	-0.12 [-0.26, 0.02]
Steger FL 2023	-3.13	4.07	15	-0.31	4.36	21	0.2%	-2.82 [-5.60, -0.04]
**Total (95% CI)**			**149**			**160**	**100.0%**	**-0.14 [-0.27, -0.01]**

Heterogeneity: $Chi^2 = 4.46$, df = 4 (P = 0.35); $I^2 = 10\%$
Test for overall effect: Z = 2.07 (P = 0.04)

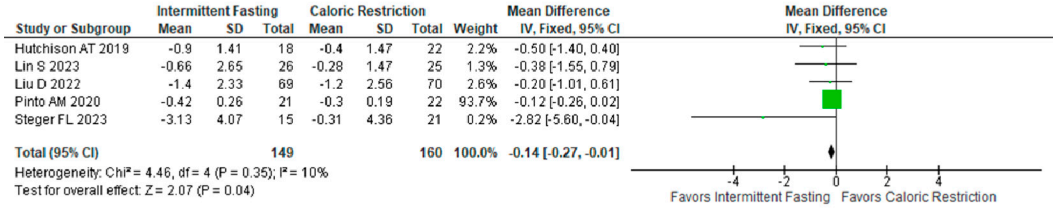

Figure S11. (A) Forest plot of the short-term effects (< 6 months) of fasting based interventions versus continuous caloric restriction on fasting insulin. (B) Forest plot of the short-term effects (< 6 months) of fasting based interventions versus continuous caloric restriction on HbA1c. (C) Forest plot of the short-term effects (< 6 months) of fasting based interventions versus continuous caloric restriction on HOMA-IR.

Table S1. Risk of bias summary: risk of bias item for each included RCT for meta-analysis according to Cochrane Risk-of-Bias Tool.

Studies	Random sequence generation (selection bias)	Allocation concealment (selection bias)	Blinding of participants and personnel (performance bias)	Blinding of outcome assessment (detection bias)	Incomplete outcome data (attrition bias)	Selective reporting (reporting bias)	Other bias
**Lin S, 2023**	Low risk. Appropriate stratified randomization.	Unclear risk. Lack of details on allocation concealment.	Unclear risk. Lack of details of blinded personnel.	Unclear risk. No mention of blinding outcome assessors.	Low risk. Dropout reasons provided.	Low risk. All outcomes reported as planned.	Unclear risk. Concerns include the study not being powered to detect small differences and lack of adjustment for multiple comparisons.
**Steger FL, 2023**	Low risk. The study used appropriate stratified randomization.	Unclear risk. Details on how allocation was concealed are not provided.	Unclear risk. Lack of details of blinded personnel.	Unclear risk. The study does not mention if outcome assessors were blinded.	Low risk. Dropout reasons provided.	Low risk. All outcomes were reported as planned, with no evidence of selective reporting.	Unclear risk. The study explicitly mentions potential biases such as self-reported dietary intake and the reduced sample size.
**Liu D, 2022**	Unclear risk. Insufficient details on random sequence generation.	Unclear risk. Insufficient details on allocation concealment.	Unclear risk. Lack of details of blinded personnel.	Unclear risk. Blinding of outcome assessment is not detailed.	Low risk. Not dropout during the study.	Low risk. All relevant outcomes were reported.	Low Risk. The study acknowledges limitations but not directly related to the experimental design.

**Thomas EA, 2022**	Low risk. The study employed appropriate randomization methods to ensure comparable groups.	Unclear risk. The details on how allocation was concealed are not provided.	Unclear risk. Lack of details of blinded personnel.	Unclear risk. The study does not specify whether outcome assessors were blinded.	Low risk. The dropout rate is reported.	Low risk. All primary and secondary outcomes were reported as planned.	Unclear risk. The study acknowledges several limitations, such as COVID-19-related disruptions and the predominantly non-Hispanic white and female sample, which could introduce bias.
**de Oliveira IR, 2021**	Low risk. The study utilized a software-based randomization process, ensuring comparable groups.	Unclear risk. Details on how the allocation sequence was concealed are not provided, leaving the potential for bias unclear.	Unclear risk. Lack of details of blinded personnel.	Unclear risk. The study does not specify whether outcome assessors were blinded.	High risk. The study had a high dropout rate (over 50%), and the handling of missing data is not adequately detailed.	Low risk. The study reports all primary and secondary outcomes as planned.	Unclear risk. The study mentions limitations such as high dropout rates and possible reduced adherence over time.
**Steger FL, 2021**	Low risk. The study used appropriate randomization methods within a parallel, randomized-controlled design.	Unclear risk. The study does not provide specific details on how the allocation sequence was concealed.	Unclear risk. Lack of details of blinded personnel.	Unclear risk. There is no mention of blinding for outcome assessors.	Low risk. The dropout rate is reported.	Low risk. The study reports on all planned outcomes, with no signs of selective reporting.	Unclear risk. The study notes limitations such as the small sample size and being a pilot study, which may introduce additional biases not fully accounted for.
**Teong XT, 2021**	Low risk. The study used appropriate randomization	Unclear risk. The study does not	Unclear risk. Lack of details	Unclear risk. There is no	Low risk. The dropout rate is	Low risk. All outcomes were	Unclear risk. The study acknowledges limitations

	methods to allocate participants to different intervention groups.	provide detailed information on how allocation concealment was maintained.	of blinded personnel.	mention of blinding the outcome assessors.	reported.	reported as specified in the study protocol.	such as a small sample size and short intervention duration.
**Pinto AM, 2020**	Low risk. The study used a computer-generated randomization process, which is an appropriate method for ensuring comparable groups.	Unclear risk. The study does not provide details on how the allocation sequence was concealed.	Unclear risk. Lack of details of blinded personnel.	Unclear risk. The study does not specify whether outcome assessors were blinded.	Low risk. The study reports on dropout rates and reasons.	Low risk. The study reports all primary and secondary outcomes as planned.	Unclear risk. The study acknowledges limitations such as the short intervention duration and specific population characteristics.
**Hutchison AT, 2019**	Low risk. The study used block randomization with appropriate stratification by BMI and age, ensuring comparable groups.	Unclear risk. The study does not provide specific details on how allocation concealment was maintained.	Unclear risk. Lack of details of blinded personnel.	Unclear risk. There is no mention of whether outcome assessors were blinded.	Low risk. The dropout rate is reported.	Low risk. The study reports on all specified outcomes, with no evidence of selective reporting.	Unclear risk. The study acknowledges limitations such as the short intervention duration and the specific population studied (overweight women).
**Sundfør TM, 2018**	Low risk. The study used computer-generated randomization with appropriate stratified sampling, ensuring	Unclear risk. The study does not detail how allocation concealment was	Unclear risk. Lack of details of blinded personnel.	Unclear risk. The study does not specify if outcome assessors were	Low risk. Dropout rates were low, and the handling of missing data was appropriately	Low risk. The study reports all outcomes as planned, with no evidence of	Low Risk. The study acknowledges limitations but not directly related to the experimental design.

	comparable groups.	maintained.		blinded.	detailed.	selective reporting.	
